# Design of Flow Systems for Improved Networking and Reduced Noise in Biomolecular Signal Processing in Biocomputing and Biosensing Applications

**DOI:** 10.3390/s16071042

**Published:** 2016-07-05

**Authors:** Arjun Verma, Brian E. Fratto, Vladimir Privman, Evgeny Katz

**Affiliations:** 1 Department of Physics, Clarkson University, Potsdam, NY 13699, USA; vermaa@clarkson.edu; 2 Department of Chemistry and Biomolecular Science, Clarkson University, Potsdam, NY 13699, USA; frattobe@clarkson.edu

**Keywords:** biosensor, flow system, biomolecular computing, noise reduction, signal processing

## Abstract

We consider flow systems that have been utilized for small-scale biomolecular computing and digital signal processing in binary-operating biosensors. Signal measurement is optimized by designing a flow-reversal cuvette and analyzing the experimental data to theoretically extract the pulse shape, as well as reveal the level of noise it possesses. Noise reduction is then carried out numerically. We conclude that this can be accomplished physically via the addition of properly designed well-mixing flow-reversal cell(s) as an integral part of the flow system. This approach should enable improved networking capabilities and potentially not only digital but analog signal-processing in such systems. Possible applications in complex biocomputing networks and various sense-and-act systems are discussed.

## 1. Introduction

Recent interest in the processing of information utilizing molecular [1,2,3,4,5,6,7] and biomolecular [8,9,10,11,12,13,14,15,16] reactions has shown promise in achieving complexity, i.e., realizing multistep signal processing to carry out computational tasks. As with most unconventional computing approaches [17,18,19] that are being studied as either alternative or complementary to modern electronics, in biomolecular computing simple digital (binary) gates and small networks of gates have been realized primarily over the past decade [8]. Furthermore, when considering enzyme-catalyzed reactions [9,15] as “gates”, progress has been made in the optimization [20,21,22,23] of the gates as well as in modifying [22,23,24,25,26,27,28,29,30,31,32,33,34,35,36,37] them with additional chemical or biochemical steps to make them better suited as “network elements” that allow for the avoidance of analog noise amplification. However, large-scale connectivity remains a challenge, due to the need to prevent cross-talk between (bio)chemical processes, which can be done [38,39,40,41] by spatial separation and “clocking” of the various processing tasks. Biocomputing systems that are capable of logically processing various biomolecular input signals have been successfully applied in binary (YES/NO) biosensors [42,43,44,45,46,47], particularly for rapid on-site analysis of injury biomarkers [48,49,50,51,52,53,54].

One approach that could potentially resolve some of these issues and enable a higher degree of complexity in gate networking has been the implementation of reaction-diffusion systems [55,56], particularly those realized in flow cells (fluidic devices) [57,58,59,60,61,62,63,64]. Recently, this approach has been utilized to realize various binary *digital* gates, including AND, OR, NAND, XOR, CNOT, etc., as well as some reversible logic gates, and small networks [59,60,61,62,63,64]. In this work, we initiate a study of the extent to which these recently developed systems can be used beyond digital signal processing to consider the *analog* nature of the input to output conversion. Recent works on potential realizations of bio-inspired information processing steps with enzymatic cascades, such as feed-forward loops [65,66] or certain memory processes [67,68,69,70,71,72], have emphasized [65,66] the importance of giving consideration to the controlled time-dependence of the analog input signal(s), and how this time dependence is reflected in the resulting time-dependence of the output. In this context, we define “analog” to mean that the actual values of the signals are considered, rather than just the specific “digital” reference values or ranges to which the signals are reduced in reference to the information in them. The primary difference is in how the noise in the signals and error-correction are handled, as well as how these signals are utilized in networking and circuit design.

In this work, we consider a simple model setup of a single-channel fluidic system with the flow of a solution containing a chromogen, ferricyanide, [Fe(CN)_6_]^3−^, that is a typical product/substrate of enzyme-catalyzed redox processes. The concentration of the chromogen along the flow channel will be denoted C(x,t), where x is the coordinate along the flow and t is the time. At the inlet, x=0, the input system is controlled to have a pulse of certain time-dependent shapes, C(0,t). At the outlet located at the end of the flow-channel, at $x=x_{c}$, the output signal’s time-dependence, $C\left(x_{c},t\right)$, is of interest. We will be interested in determining to what extent the output signal is able to respond to different input pulse shapes. However, the actual optical measurement is carried out in a cuvette into which the outflow is fed. The concentration of the chromogen in the cuvette, S(t), at time t, depends on the cuvette properties and on the inflow rate into it for all times up to t. The details will be described in the theoretical section (Section 3). The experimental setup will be presented in the experimental section (Section 2). We will consider the rectangular and triangular input pulses, as well as the situation for the former (rectangular-pulse) experiment when part of the chromogen is consumed by an enzyme-catalyzed reaction (described in Section 4), utilizing enzyme diaphorase. This enzyme will be immobilized in a small section of the flow channel, with its function activated by the addition of the required substrate chemical, β-nicotinamide adenine dinucleotide reduced (NADH). Results and discussion are presented in Section 4. Section 5 offers concluding remarks.

**Figure 1:**
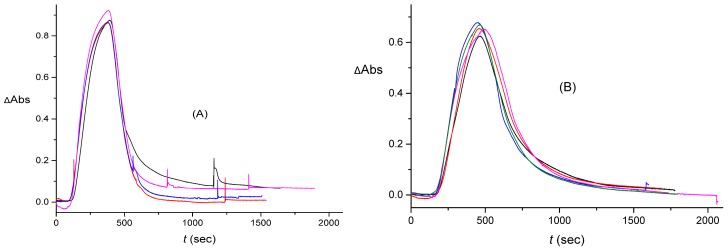
Example of the output signal, S(t), measured as the change of the absorbance (with respect to the background) in the cuvette as a function of time. (**A**) Four different experiments for the rectangular input pulse; (**B**) Five experiments for the triangular input pulse. Both data sets were obtained without the enzymatic reaction. The optical absorbance was measured at λ = 420 nm, corresponding to λ_max_ in the absorbance spectrum of [Fe(CN)_6_]^3−^.

In the rest of the introduction, we will highlight and illustrate the challenges involved in data interpretation for such experiments. In Figure 1 we present two sets of results representing both the rectangular and triangular input pulses. Both initial-pulse types, C(0,t), were 330 s in duration. Initially, it takes the pulse 100 s to reach the cuvette. This delay is seen as the initial, approximately zero signal region in the panels of Figure 1. However, once the signal, S(t), picks up, it lasts noticeably longer than 330 s, with a long tail. We observe significant noise expressed as fluctuations in the signal values within each data set and between the various experiments, as well as the presence of spikes likely attributable to bubbles. Some of the noise (and the bubbles) that is present is due to imprecise control of the pulse shape on injection.

As will be shown in Section 4, all the aforementioned sources of noise and uncertainty in the signal are actually much more pronounced in the input into the cuvette, $C\left(x_{c},t\right)$, The measured signal S(t), which is proportional to the change in the absorbance at 420 nm, involves a certain averaging due to mixing in the cuvette, as modelled in Section 3. The tradeoff, however, is that the shape of the output signal S(t) in Figure 1 does not looks as that dissimilar for the rectangular and triangular inputs. As a result, it may be deduced that S(t) is not well suited for analog information/signal processing, because it does not clearly reflects the original pulse shape, which, as will be argued in Section 4, should not be significantly distorted on transport in the channel, from C(0,t) to $C\left(x_{c},t\right)$. The signal S(t) can only be used as a digital overall-intensity- or peak-intensity-based measure, exemplified as Yes/No (On/Off), for the presence/absence of the signal.

Our goal will be to explore to what extent the averaging due to mixing in the cuvette, which produces the output signal, S(t), can be “de-convoluted” to yield back $C\left(x_{c},t\right)$. We will establish that this can be done to a good approximation. However, then the original larger noise level in $C\left(x_{c},t\right)$ will be revealed, and will require a different averaging to see the expected analog signal shapes. We will also argue that properly designed mixing cells can be useful in enabling larger number of steps in consecutive networking for digital information processing with such flow systems.

## 2. Experimental Setup

### 2.1. Materials and Reagents

The chemicals used in this system included but were not limited to an enzyme that was immobilized in a section of the flow channel: diaphorase from *Clostridium kluyveri*, EC 1.8.1.4; β-nicotinamide adenine dinucleotide reduced (NADH) dipotassium salt; H_2_O_2_ (30% wt. in H_2_O); glutaric dialdehyde; poly(ethyleneimine) solution (PEI) (average Mw ca. 750,000); 2-amino-2-hydroxymethyl-propane-1,3-diol (Tris-buffer); and ferricyanide K_3_[Fe(CN)_6_] (extinction coefficient 1040 M^−1^·cm^−1^). The chemicals listed above were purchased from Sigma-Aldrich (St. Louis, MO, USA) and used as supplied. Ultrapure water (18.2 MΩ∙cm) from NANOpure Diamond (Barnstead, Lake Balboa, CA, USA) source was used in all of the experiments. Flow cells (µ-Slide III 3 in 1 Flow Kit; ibidi GmbH, Martinsried, Germany) were used for the biocatalytic reactions. The overall length of the flow cell’s channel is 45 mm. The main reacting volume of the enzymatic flow cell is 23.73 mm long by 3 mm wide, with a depth of the channel being 0.4 mm. The small channel that feeds into this main reacting volume is 10 mm long; with a width of 1 mm and a depth of 0.4 mm. Additional comments on chemicals used are given in Supplementary Materials.

### 2.2. Flow System Design

**Figure 2:**
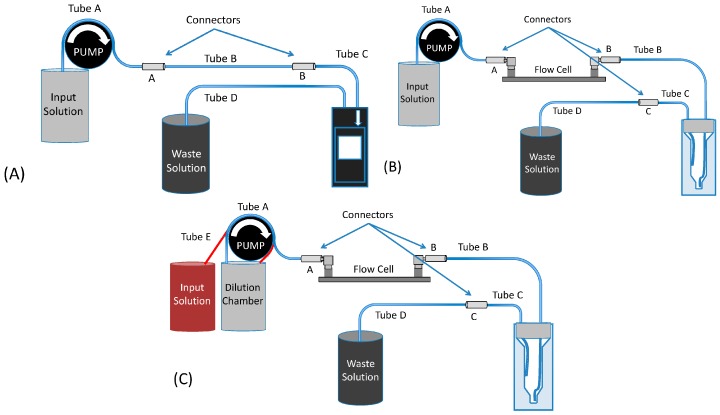
Flow system design. (**A**) The input solution is pumped through Tube A (length *l* = 39 cm, diameter *d* = 0.5 mm), through Tubes B (*l* = 22.5 cm, *d* = 1.0 mm) and C (*l* = 10 cm, *d* = 1.0 mm), and into the commercially available (shown here) flow-through cuvette, exiting via Tube D (*l* = 50 cm, *d* = 1.0 mm); (**B**) A flow cell with immobilized enzyme was added into the system, with the lab-made cuvette used (shown here). Note that the cell is rather small (see Subsection 2.5) in all its dimensions, and is exaggerated here. Tubes A, B and C parameters here are the same as before (but they are differently connected). Connector C provides the mechanical stability needed to control the positioning of the outflow tube C and thus keep the volume of the liquid in the cuvette constant; (**C**) A dilution chamber was added to allow the input of a “triangular” pulse. The input solution is pumped through Tube E (*l* = 39 cm, *d* = 0.5 mm) into the dilution chamber. Simultaneously, Tube A is used to pump the solution out of the dilution chamber via the same pump.

When constructing the flow system, sketched in Figure 2, every possible effort was made to create a uniform system that would isolate the differences in the cuvettes for experiments with otherwise similar flow rates. In practice, this was accomplished by utilizing a uniform set of tubing and connectors in conjunction with a peristaltic pump that is able to provide a steady flow of solution. All experiments that included a flow cell did so with it being functionalized with the enzyme, as described in Section 2.5, even when the enzyme-catalyzed process was not ongoing (because NADH was not introduced in solution).

In Figure 2, the flow systems that were used are sketched. The first system, Figure 2A was the most straightforward setup, with the pump connected to a commercially purchased flow-through cuvette. The same system was then modified for the integration of a tunable-volume cuvette, which was lab-made. This cuvette was designed to shorten the tail in the time dependence of the output signal, cf. Figure 1. The system was then re-configured to accept the addition of an enzyme-functionalized flow cell, Figure 2B. The µ-Slide III 3in1 Flow Kit cell contained diaphorase, 8.55 U, that was immobilized utilizing a Schiff base reaction. The final configuration change, Figure 2C, was the addition of a controlled dilution system. This system enables the pulse to be applied in a manner that allows for both an increasing and decreasing concentration over time, as addressed in Section 2.3. The resulting flow rate values into the cuvette varied in the range of 176 to 210 μL/min.

The output signal was measured optically as the change in the absorbance of [Fe(CN)_6_]^3−^ at 420 nm in the cuvettes in real time by utilizing a UV-2450 UV-Vis spectrophotometer (Shimadzu, Tokyo, Japan). Photographs of the experimental setup are given in Supplementary Materials.

### 2.3. Control of the Input Pulse

A peristaltic pump (MINIPULS^®^ 3, Gilson, Middleton, WI, USA) with a head diameter of 6.5 cm was utilized to control the velocity of the chromogen solution applied to the system. Our rectangular-shaped input pulses were 0.5 mM [Fe(CN)_6_]^3−^ for the initial experiments, and were then increased to 1.0 mM for all the subsequent experiments. In the initial experiments, the 0.5 mM input pulses were applied to the commercially purchased as well as to the tunable, lab-made flow cuvette. After the initial testing on the flow through cuvettes, the tunable, lab-made cuvette was used exclusively in the flow system outlined in Figure 2B,C. For the “triangular” pulse that was also passed through the system, Figure 2C, the pulse shape was approximated by first pumping 1.0 mM [Fe(CN)_6_]^3−^ into the dilution chamber shown in Figure 2C. This results in an approximately linear increase in the outflow concentration from that chamber into the system. To obtain an approximately linearly decreasing concentration, after the peak point of the triangular pulse, solution without [Fe(CN)_6_]^3−^ was pumped into the dilution chamber. Finally, in the enzymatic tests, the 1.0 mM [Fe(CN)_6_]^3−^ rectangular pulse was supplemented with NADH (1.0 mM) which is oxidized by diaphorase to yield NAD^+^ while reducing [Fe(CN)_6_]^3−^ to [Fe(CN)_6_]^4−^. This allowed a portion of the rectangular pulse to be enzymatically consumed.

### 2.4. Design of the Cuvettes

**Figure 3:**
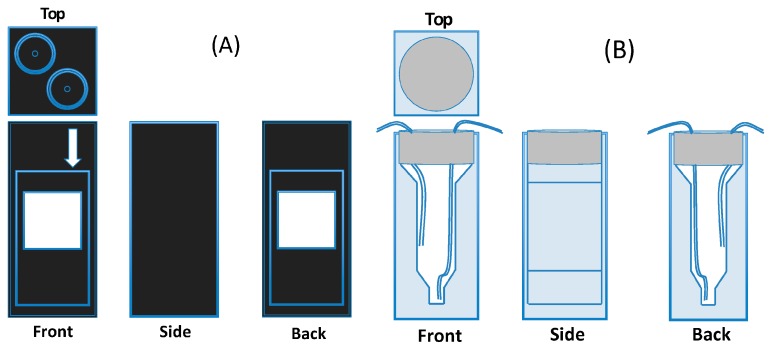
(**A**) The commercially available flow-through cuvette. This cuvette utilizes two threaded connectors at the top of the chamber to keep the vessel pressure sealed. The inflow pipe, marked by the arrow on the sketch, is plumed to feed the visible chamber from the bottom, with the outflow being plumed into the top of the cuvette’s chamber. The internal volume of the cuvette is $0.422{\text{ cm}}^{3}$; (**B**) The lab-made tunable cuvette. It was made by utilizing disposable plastic cuvettes for the basic structure and is sealed using a rubber septum. The septum allows for the inflow and outflow pipes to have their depth manually set to tune the volume of the trapped air and thus also the volume of the liquid in cuvette.

A commercially available flow-through cuvette is sketched in Figure 3A. This cuvette has an internal volume of 0.422 cm^3^ and an optical pathway of 1 cm. The flow of solution enters the cuvette through an inlet plumbed into the bottom of the optical chamber and exits the cuvette through an outlet that is plumbed into the top of the optical chamber. The lab-made cuvette, Figure 3B, has a tunable volume of liquid in it. The lab-made cuvette was constructed from a standard disposable cuvette—with a 1 cm optical pathway—that was sealed with a rubber septum. The seal formed between the rubber septum and the tubing is a pressure fit. Similarly to the commercially available cuvette, the inflow pipe’s depth is set to the bottom of the cuvette. The outflow pipe allows for the ability to tune the liquid volume in the cuvette. This is done by varying the depth of the outflow pipe to control the volume of the trapped air in the cuvette. After the initial testing, liquid volume values of 0.300 cm^3^ and 0.474 cm^3^ were used. Photographs of the cuvettes are given in the Supplementary Materials.

### 2.5. Immobilization of the Enzyme

Before any experimental data were measured, the flow cell in which enzyme is immobilized, and which is part of the flow system (Figure 2), was flushed with H_2_O_2_ for a minimum of 3 h, and then washed with a minimum of 10 mL of deionized water. These cleaning steps aimed at removing any physically adsorbed PEI from the internal surface of the flow cell as well as any enzymes from previous experiments while preparing the surface for adsorption of PEI. Then, the flow cell was treated with a PEI solution (2% v/v) for 1 h and thoroughly washed with 5 mL of deionized water, resulting in physical adsorption of PEI on the internal polystyrene surface and providing the amino groups needed for the enzyme immobilization. Then, the amino-functionalized surface was reacted with glutaric dialdehyde (5% v/v) for 1 h; after that, the surface was washed with 5 mL of deionized water to remove non-reacted glutaric dialdehyde. The solution containing the enzyme diaphorase (8.55 U/flow cell) was then reacted with the flow cell that had been activated with glutaric dialdehyde for 1.5 h. Glutaric dialdehyde operated as a linker producing Schiff-base covalent bonds with amino groups of PEI and the enzyme. After the coupling of diaphorase to glutaric dialdehyde, the cells were thoroughly washed with Tris-buffer (0.1 M, pH 7.1) to remove non-reacted enzymes from the cells. Additional details on the immobilization of the enzyme, and flow-cell photograph and schematic are given in the Supplementary Materials.

## 3. Theoretical Considerations

### 3.1. Flow in the Channel

**Figure 4:**
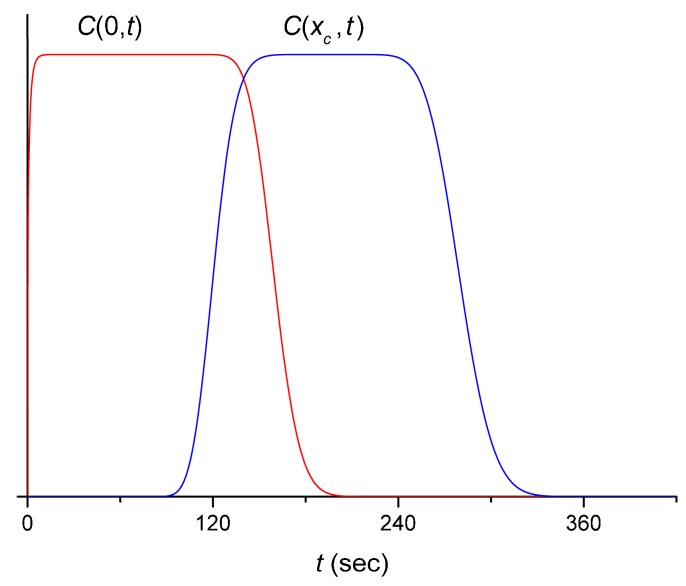
Diffusional spreading of the initially approximately rectangular pulse (arbitrary units) of 170 s duration (red curve), calculated from Equation (1). Here the velocity of the flow was taken as 0.1 cm/s, which is comparable to the experimental value, and we used a representative value $D=7\;\times 10^{-6}\;{\text{cm}}^{2}/s$. The blue curve represents the time-dependence of the pulse at the end of the channel, taken 12 cm long.

The initial pulse, C(0,t), is modified on traversing the channel, to $C\left(x_{c},t\right)$, and then further modified in the cuvette to S(t). In this subsection we consider the former process, and we focus on the degree to which the diffusion of the chromogen alters the shape of the pulse. This assumes that convective (hydrodynamic) mixing in the narrow tubes located in the main flow channel is negligible [73]. Specifically, we ignore the possible effects of the pump, connectors or junctions. Thus, for simplicity we assume that the flow is uniform and the flow velocity, v, in the entirety of a “representative” flow channel can be considered constant to estimate the diffusional spreading of the pulse, which is then described by:

(1) $$\frac{dC}{dt}=D\frac{d^{2}C}{dx^{2}}-v\frac{dC}{dx}$$

where $D=7.26\;\times 10^{-6}\;{\text{cm}}^{2}/s$ is the diffusion constant of ferricyanide [74]. For our values of v in the wider tubes (slower flow) varying in the range of 0.09 to 0.12 cm/s, the length of the tubes totaling from 80 to 124.5 cm forming the flow channel, and the pulse duration of 330 s, the diffusional spreading of the pulse was found to be relatively small. This is illustrated by solving Equation (1) numerically: see Figure 4, where we took typical parameters for a slower-flow (wider) tube and assumed an approximately rectangular input pulse, which is in itself already somewhat spread out by the injection process.

### 3.2. Measurement in the Cuvette

The cuvette, especially the lab-made one with flow reversal, can cause significant convective mixing. This is similar to the well-mixed reactors [75] used in chemical engineering, yet in our realization, no chemical reactions are present in the cuvette. We will assume that the chromogen is well-mixed in the cuvette at all times of relevance to the measurements, and therefore its outflow from the cuvette is approximately proportional to its concentration, which is in turn proportional to the measured signal, S(t). We thus assume that:

(2) $$\frac{dS}{dt}=vg_{1}C\left(x_{c},t\right)-vg_{2}kS$$

where $g_{1,2}$ include geometrical factors and depend on the dimensions of the cuvette, notably, on the volume of the liquid in it, on the inflow and outflow pipes, and the cell’s internal structure (cf. Figure 3).

Since the actual measured signal, the change in the absorbance, ΔAbs, is only *proportional* to S(t), then as long as we do not consider (define) the precise value of S(t), but only use arbitrary units for it and in fact also for $C\left(x_{c},t\right)$, we can absorb all the constants in the definition of S and the proportionality constant. We then use a simplified equation:

(3) $$\frac{dS}{dt}=C\left(x_{c},t\right)-KS$$

with the new proportionality constant, *K*, expected to be approximately constant for each given type of cuvette (the precise geometry of its interior and the liquid inside) and fixed inflow velocity.

When a well-defined pulse reaches the cuvette, there is an initial fast increase in the signal, see Figure 1, due to the first term in Equation (3). However, after the pulse duration time, there can be an extended decaying “tail” in the signal due to the second term in Equation (3). This tail is approximately proportional to $e^{-Kt}$ and can be quite long-duration, obscuring the original pulse shape, as described in Section 4.

## 4. Results and Discussion

### 4.1. Effect of the Cuvette on the Signal

**Figure 5:**
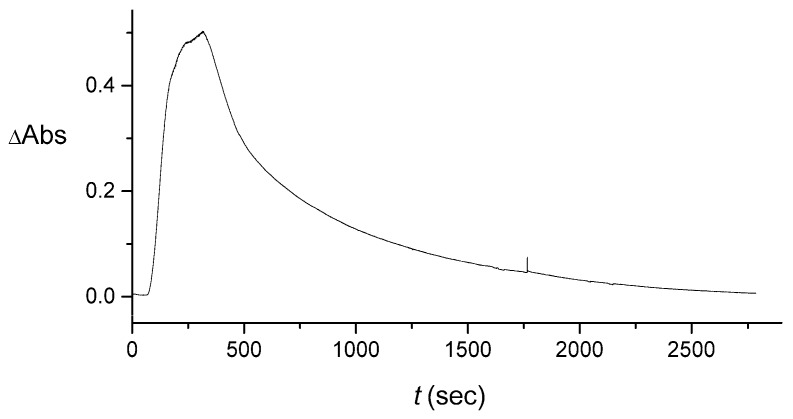
Example of the measured output signal, S(t), produced with a commercially available cuvette, for a rectangular pulse. The numerically estimated decay time of the tail, 1/*K*, cf. Equation (3), by data fitting for this system is nearly four times longer than the value for the systems shown in Figure 1.

As explained earlier, the constant *K*, which determines the tail in the signal, S, is expected to depend on the geometry of the cuvette and the flow velocity in it. Hence we can potentially manipulate the decay of S by taking cuvettes of different designs. As alluded to in Section 2, we initially used a *commercial cuvette*, in the setup shown in Figure 2A that has a relatively long tail, has no flow reversal, but might have internal structure leading to mixing that was neither controllable nor specified. A signal produced with this cuvette is shown in Figure 5. The flow rate into the cuvette was 176 μL/min, and its volume was specified earlier, 0.422 cm^3^. The fitted *K* value was $2.0\times 10^{-3}$ s^−1^. It should be noted that in these trials, the input pulse was rectangular. The error bars in the fitted K values here and below were up to 2% of the given values, and when selecting the “tail” for fitting, it was determined that it should incorporate the time it took for the pulse to reach the cuvette plus the input time duration and an extra small-duration broadening due to diffusion, as further commented on in the next subsection.

After some initial tuning of the *lab-made cuvette* design in the same setup, we switched to the setups shown in Figure 2B,C. Measured output signals with a relatively short tail are illustrated in Figure 1A,B, which correspond to Figure 2B,C, respectively, with a lab-made cuvette of volume 0.474 cm^3^, inflow rate 210 μL/min, and the fitted *K* value (averaged over the measured four rectangular, five triangular, and also two rectangular-reacted pulses described later) was $8.0{\times 10}^{-3}$ s^−1^. Another, more limited set of data (show later) was measured after the liquid volume in the lab-made cuvette was reduced to 0.300 cm^3^, with the inflow rate somewhat varying: 195 μL/min for the setup of Figure 2B, for rectangular and rectangular-reacted pulses, and 184 μL/min for Figure 2C, for the triangular pulse. The average value of *K* for this cuvette was $7.6\times 10^{-3}$ s^−1^.

The *lab-made cuvettes* not only reduced the tail, but also allowed us a preliminary verification of the following expectation. After the flow-velocity, *v*, dependence in Equation (2), as well as the potential dependence on the cell volume and geometry via $g_{1,2}$ were absorbed into the definition of *K* in Equation (3) and also into the arbitrary units of the output signal $C\left(x_{c},t\right)$, there might be some residual cell-volume, *U*, and flow-velocity dependence of *K*. If this dependence correlates to the variation of the mixing rate in this flow-reversal cell, then there are arguments [75] that suggest that the variation of *U* and of $v^{3}$ yield the same result. Interestingly, the ratios of the two lab-made volumes just quoted are approximately 1.6, and the inverse ratios for the flow velocities cubed are 1.3–1.5. The ratio of the average *K* values is nearly 1, and we comment that within each data set the fitted values of *K* are spread by about 20%, which reflects the fact that the single-exponential-tail decay model is approximate. This is further discussed in the next subsection.

### 4.2. Extraction of the Signal Produced by the Flow System

As illustrated in Figure 1, the “averaging” that is a result of mixing in the cuvette makes the measured signals that are produced by the initially rectangular and triangular pulses more or less similar. Obviously, this effect of the cuvette obscures the shape of the pulse. We can remove the effect of the cuvette numerically, to obtain $C\left(x_{c},t\right)$ from S(t) by using Equation (3). This is done by first fitting the parameter *K* from the large-time tails of the measured signals. Then the full signal $C\left(x_{c},t\right)$ is calculated from Equation (3).

Numerical data-fitting for the decaying tails was done for several measured systems. The result of this in reference to *K*, illustrates that *K* is somewhat dependent on the choice of the cutoff, past which the shape of the pulse fed into the cuvette no longer matters, because it decreases to practically zero. Because the data are noisy and the tail shows a fast (exponential) decay, this cutoff is not trivial due to its effect on the largest values of S(t) that are included in the fitting. When considering this in conjunction with the variations present between experiments, spread in the fitted values of *K* is estimated to be up to 20%. However, the expectation that when the same cuvette and flow-rate into it are involved, *K* should be the same is confirmed in that the average values of *K* quoted earlier suffice to approximately remove the tails for the appropriate data sets. This is illustrated below, where we discuss the results for the signals, $C\left(x_{c},t\right)$ (in arbitrary units) that have been calculated.

**Figure 6:**
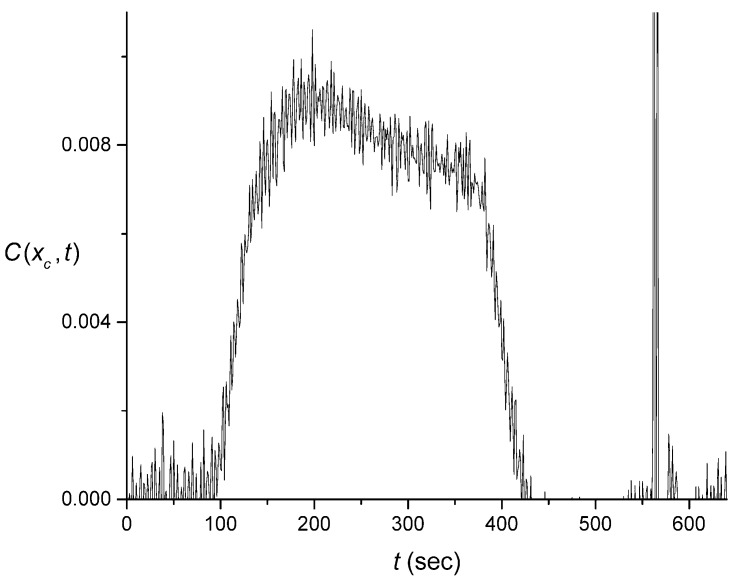
Example of a rectangular input pulse $C\left(x_{c},\;t\right)$ generated from one of the measured output signals, S(t) , shown in blue color in Figure 1A, without any signal smoothing/averaging. The erratic variation at ~565 s is likely an effect of a bubble in the input channel.

Removing the effect of the averaging due to mixing in the cuvette exposes a large level of noise that is inherently present in the flow-system signals. This noise occurs on short time scales as well as globally. The latter is visible as distortions of the signal. The “fast” noise is so large that the latter distortion is not clearly discernable until smoothing/averaging of the former is performed. A reconstructed pulse without any signal smoothing is illustrated in Figure 6. As mentioned earlier, the noise that is exposed during the reconstruction of the pulse, was initially obscured by internal mixing that occurred in the cuvette during the measurement of the signal.

### 4.3. Numerical Averaging to Decrease the Noise Effects

As illustrated in Figure 6, the fast noise visible in the signal represents fluctuations over time scales ranging from seconds to a small fraction of a minute. One approach that may be used to extract a signal that is smooth on these time scales involves the averaging of S(t) over a certain window of points before calculating $C\left(x_{c},t\right)$. We obtained the averaged $\bar{S}\left(t\right)$ by using S(t) values averaged over the interval of ±20 s around *t*. An attempt to average $C\left(x_{c},t\right)$ after its extraction was found to require an approximately double time interval, which might obscure some of the shape-distortion features. Averaging over a smaller time interval, did not effectively remove the noise.

**Figure 7:**
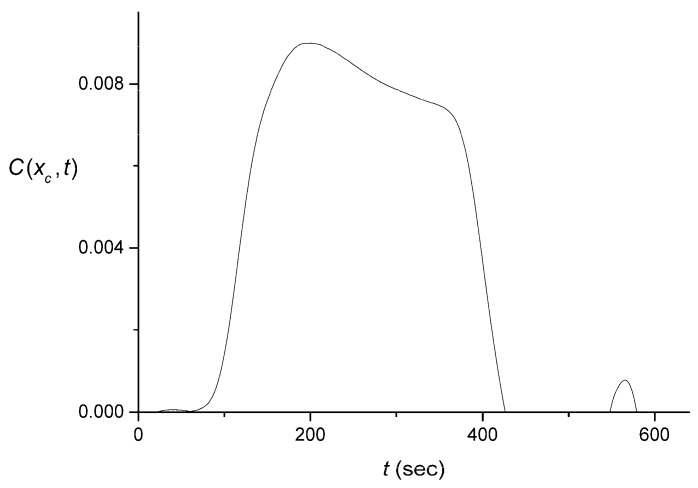
Example of a rectangular input pulse $C\left(x_{c},\;t\right)$ generated from the averaged output $\bar{S}\left(t\right)$ , which was in turn obtained for one of the signals presented in Figure 1A, the same one that was used for Figure 6.

**Figure 8:**
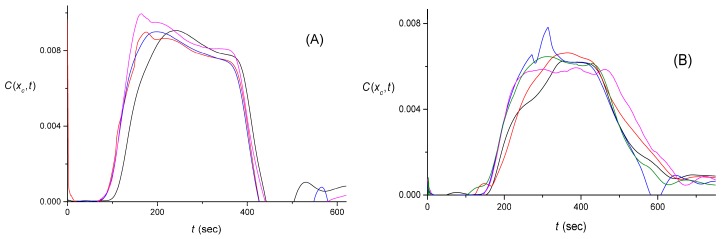
The input pulses, $C\left(x_{c},\;t\right)$, in arbitrary units, generated from the signals, S(t), shown in Figure 1, after the latter were smoothed out as described in Section 4.3. The individual pulses are color coded with Figure 1. (**A**) Four different rectangular input pulses; (**B**) Five different triangular input pulses. Both sets were obtained without the enzyme-catalyzed process being activated.

The pulse shown in Figure 6 is replaced, after carrying out the described averaging, by the one shown in Figure 7. We performed the same averaging for all the data sets measured with the larger-volume lab-made cuvette. The original signals for the rectangular and triangular pulses are shown in Figure 1. After the averaging that has been described above, these three data sets yielded $C\left(x_{c},t\right)$ shown in Figure 8.

**Figure 9:**
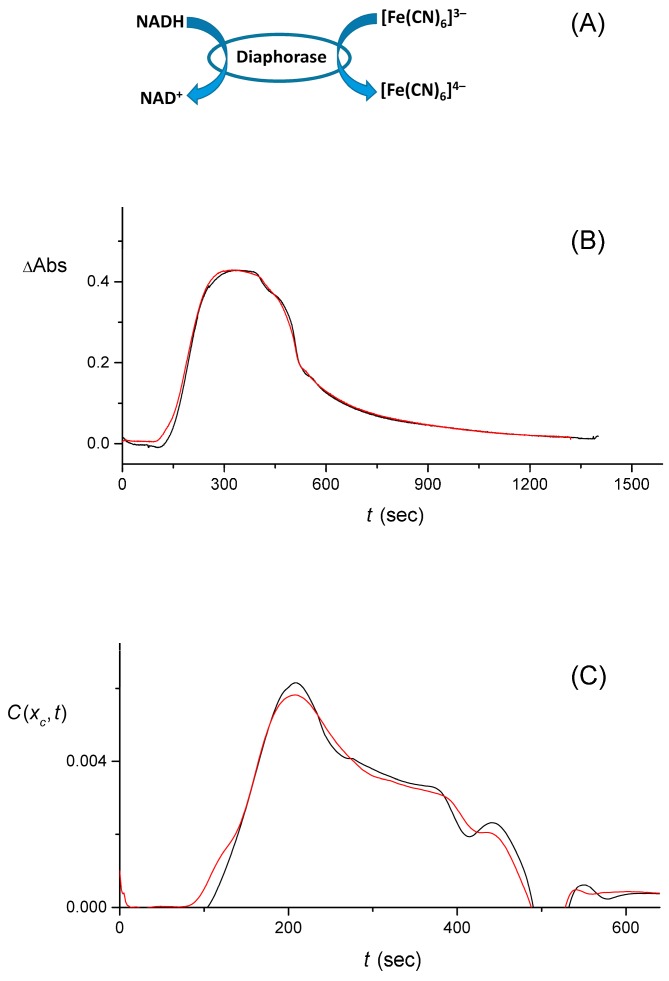
(**A**) Schematics of the ferricyanide, [Fe(CN)_6_]^3−^, being reduced by NADH, in a process biocatalyzed by diaphorase. The reaction products are β-nicotinamide adenine dinucleotide (NAD^+^, oxidized form) and ferrocyanide, [Fe(CN)_6_]^4−^. Note that the optical absorbance (λ_max_ = 420 nm) decreases upon reduction of [Fe(CN)_6_]^3−^; (**B**) Two experimentally measured signals, S(t), for a rectangular pulse with the biocatalytic process ongoing (activated by adding 1 mM NADH); (**C**) Shows the numerically reconstructed input pulses, $C\left(x_{c},\;t\right)$ , generated from smoothed-out S(t) .

**Figure 10:**
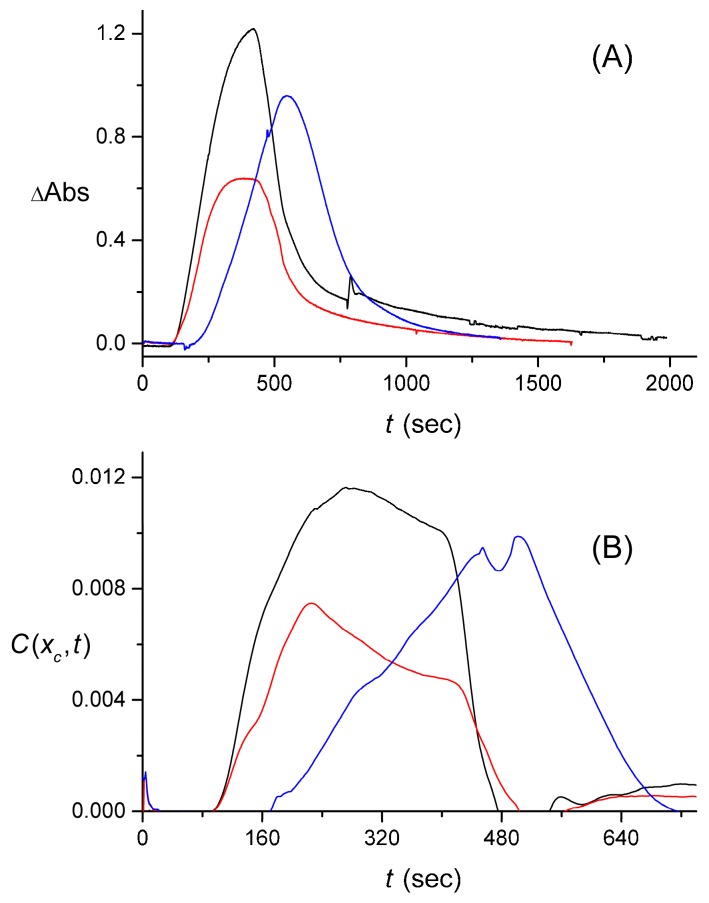
Experimental data measured using a cuvette of a relatively smaller volume. (**A**) Signals, S(t), generated from rectangular (black) and triangular (blue) pulses without the enzymatic process, and from a rectangular pulse (red) with the enzymatic process ongoing; (**B**) The reconstructed input pulses, $C\left(x_{c},t\right)$ , shown with the same color coding, obtained from the three measured signals, S(t) , after the latter were smoothed out.

Another set of data that was obtained via the use of the larger-volume lab-made cuvette, involved the measurement of a signal with a reacted (catalyzed by the enzymatic process) rectangular pulse. Figure 9A offers a schematic of the enzymatic process. The two measured signals, S(t), are shown in Figure 9B. After averaging the signal as described earlier, these two data sets yielded $C\left(x_{c},t\right)$ shown in Figure 9C. Finally, the results obtained from the data measured with the smaller-volume lab-made cuvette (one data set for each input pulse shape and for the case with the enzymatic process) are presented in Figure 10.

### 4.4. The Quality of the Pulse Shape Reconstruction

Note that when more than a single data set was taken, we did not average over them. This was done because we are interested in single-signal processing and “connecting” the processing steps consecutively, as described in Section 4.5 and Section 5. Qualitatively, the reconstructed pulse shapes, $C\left(x_{c},t\right)$, in Figure 7, Figure 8, Figure 9 and Figure 10 for each individual signal are similar to what is expected. This correlation supports the approach described above, focusing on the use of Equation (3), in removing the tail in the signal S(t) after it is smoothed out by averaging, using a single representative value of *K* for a particular cuvette (and flow rate).

Averaging removes some of the noise in the signal, and this partially includes the splashes due to bubbles and the fluctuations on time scales of a fraction of a minute. However, the resulting pulse shapes are also distorted from the originally expected rectangular or triangular shapes. In fact, in all the pulses that are expected to be rectangular with perhaps small broadening by diffusion, we not only observe random-looking distortions, but also some decay as a function of time. A similar feature can be noticed for triangular pulses. This effect could be attributable to the notion that the chromogen is likely mixed and accumulated in parts of the experimental setup other than the cuvette. Therefore, mixing effects—of the type defined by Equation (3)—that result in accumulation followed by exponential release, occur to some extent in other flow-system components and represent a limitation of the experimental design. With the aforementioned observation in mind, we can then conclude that the time dependence of the signal shape can be semi-quantitatively recovered at best with the time resolution of 1–2 min.

Consideration of the data for the cases with the enzyme-catalyzed process present, seem to suggest that effects of this process are two-fold. First, the signal is generally decreased, which is expected because part of the chromogen is biocatalytically reduced and does not contribute to the output signal. Yet there is also a systematic difference in the way the rectangular shape decreases with time for the reaction vs. no-reaction experiments, see Figure 9C vs. Figure 8A, and Figure 10B, red vs. black curves. However, the precise quantification of the effects of the enzymatic reaction is not entirely practical because the data are too noisy and not conclusive.

### 4.5. Discussion of Signal Processing and Networking

The observations summarized in Section 4.4 explain why flow systems such as the type described here have only been used for “digital” signal processing and only when combined in rather small networks. When considering the large differences in cuvette-averaged output signals at certain “gate” times that allow for the ability to resolve the **1** vs. **0** reference output signal values, the limitation to the “digital” signals is clear. In the case of analog signal processing, output signals with much less noise and with good time-resolution of the noise-eliminated time-dependence on time scales of the original input variations are needed. In the studied system, however, the noisy time resolution was at best possible on time scales of a couple of minutes, whereas the whole input pulse duration was typically only about 5.5 min.

We point out, however, that the devised averaging procedure did allow for a semi-quantitative pulse-shape recovery. More generally, averaging of the type that occurs due to the cuvettes can be beneficial for digital signal processing. If such averaging is also fast enough to cover only time-scales of the noise (±20 s in our case), it can enable analog processing with better system design. Out of all the possible physical or chemical processes that may be implemented to carry out averaging, the simplest one might be to devise well-mixing (which can be done for example by flow-reversals [75]) flow-through cells (similar to our cuvettes) to be inserted in the flow channel. The desirable flow-cells should have 1/*K* of the order of seconds, rather than our values of ~125 s for the lab-made and 500 s for the commercial cuvette. Generally, this criterion is suggested by that, typical time scales of signal inputs in biosensing and biomedical applications are seconds to minutes [76], so averaging over such time intervals is already allowed for and expected. We also note that the general flow properties and the flow-cell mixing/signal averaging capabilities depend on the hydrodynamics of the flow, and therefore the use of microfluidic devices [77] might offer new pathways to improved networking.

## 5. Conclusions

In summary, the idea proposed in this work as suggested by our results, is to introduce physical “noise averaging” by adding mixing cells, to mimic our computational averaging that enables removal of noise and enables the identification of the pulse shapes. The cuvettes that are part of this and other experimental setups, accomplish a form of averaging but on time scales too large to be useful. With the implementation of properly designed cells for noise reduction, it should be possible to advance the use of flow systems to multi-step consecutive connectivity in order to increase the complexity of the digital signal processing, as well as the inherent networking capabilities. These advances have the potential to also enable analog signal processing. Overall, the present research offers important conclusions that are useful for various biomolecular computing systems [59,60,61,62,63,64] as well as biosensors with the flow-design [78].

Biochemical computing and logic gate systems based on biomolecules have the potential to revolutionize the field of biosensors and bioactuators. Interfacing these biocomputing elements with sensing processes would allow multi-signal analysis followed by biochemical processing of the data, giving a final digital (YES or NO) analytical answer. Such YES/NO information also allows direct coupling of signal processing with signal-responsive materials and chemical actuators to offer the possibility of a closed-loop sense-and-act operation. Biochemical networks can offer robust error-free operation upon appropriate optimization of their components and interconnections. The whole research area may benefit from shifting the interests and motivations from pure computational goals, which are not easily realizable at the present level of technology, to designing the interface between biochemical/biological systems and electronics/materials for logic processing of signals within presently available systems of limited complexity. Technological realization of the information processing systems in flow devices allows for “clocking” (temporal control) as well as spatial separation of the various steps of multistage biochemical processes, thus providing novel options for their sophistication and functional flexibility.

## Supplementary Materials

The following are available online at http://www.mdpi.com/1424-8220/16/7/1042/s1. Additional comments on chemicals used; additional details on the immobilization of the enzyme, and flow-cell photograph and schematic; photographs of the experimental setup; and photographs of the cuvettes.
