# Supplementary material for: Design of Flow Systems for Improved Networking and Reduced Noise in Biomolecular Signal Processing in Biocomputing and Biosensing Applications
Source: Sensors (Basel). 2016 Jul 5;16(7):1042. doi: 10.3390/s16071042 (PMC4969838; doi:10.3390/s16071042)
Supplement: Supplementary file 1 [file sensors-16-01042-s001.pdf]

Supplementary Materials: Design of Flow Systems for Improved Networking and Reduced Noise in Biomolecular Signal Processing in Biocomputing and Biosensing Applications

Arjun Verma, Brian E. Fratto, Vladimir Privman and Evgeny Katz

1. Chemicals

Chemicals used for the immobilization of the enzyme and the preparation of the system included H_2O_2 (30% wt. in H_2O), glutaric dialdehyde, poly(ethyleneimine) solution (PEI) (average Mw ca. 750,000).

2. Immobilization of the Enzyme in the Flow Cell

The following procedure was originally published in [1,2]. Minor adjustments in the experimental steps were performed in order to optimize the system for our specific experiment. Before any experimental measurements were carried out, the flow cell was flushed with concentrated H_2O_2 (30% wt. in H_2O) for 3 h to remove residual PEI that was adsorbed on the cell surface from previous experiments. The cell was then washed with at least 10 mL of deionized water. These cleaning steps aimed at removing residual chemicals from previous experiments and preparing the cell surface for adsorption of PEI.

Then, the flow cell was treated with a PEI solution (2% v/v) for 1 h and afterwards, thoroughly washed with 5 mL of deionized water, resulting in physical adsorption of PEI on the inner polystyrene surface and providing the amino groups needed for the enzyme immobilization. The amino-functionalized surface was then reacted with glutaric dialdehyde (5% v/v) for 1 h. After that, the surface was washed with 5 mL of deionized water to remove non-reacted glutaric dialdehyde.

The enzyme solution was reacted for 1.5 h with the flow cell that had been activated with glutaric dialdehyde, and then the cell was thoroughly washed with Tris-buffer (0.1 M, pH 7.1) to remove non-reacted enzyme from the cell. The following enzyme concentration was used for the modification of flow cell: 8.55 U of diaphorase per flow cell. The flow cell device with the immobilized enzyme demonstrated reproducible performance for at least two days allowing pumping of the input solutions over long period of time, thus proving stable immobilization of the enzyme and preserving its biocatalytic activity.

3. Photographs and Schematic

A photograph of the enzymatic flow cell and its schematics are shown in Figures S1 and S2, respectively. Photographs of an experimental setup (with the flow cell) are shown in Figures S3 and S4, while the two flow-through cuvettes are shown in Figures S5 and S6.

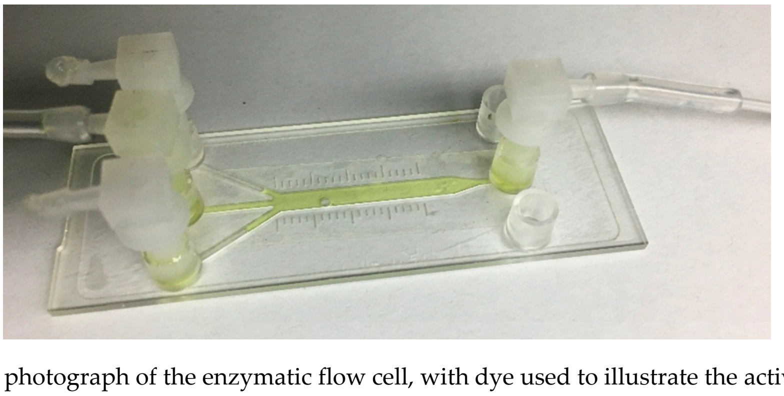

Figure S1. A photograph of the enzymatic flow cell, with dye used to illustrate the active chamber.

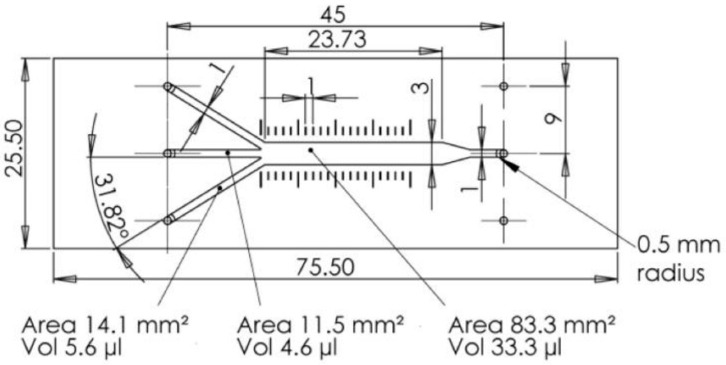

Figure S2. The schematics of the flow cell used in the enzymatic experiments. The dimensions noted in the schematic are in millimeters. The internal total channel volume of the flow cell is 60 μL, with the height of all the channels being 0.4 mm and the width of the channels thin/thick being 1 mm and 3 mm, respectfully. (Adopted from the company web-site with permission: http://www.autom8.com/biomicro-SlideIII-3in1.html, last visited 22 April 2016.).

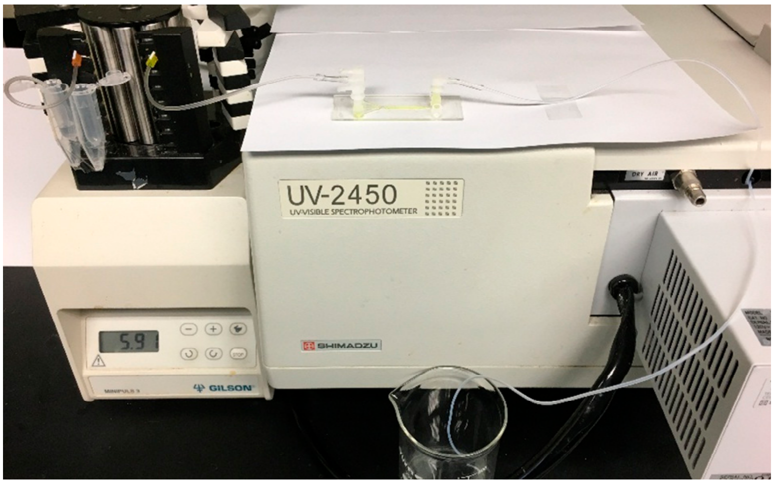

Figure S3. The experimental setup that was used to analyze the process of signal transduction. The flow of solution was controlled by a Gilson MINIPULS® 3, which was used to push solution through the enzymatic flow cell and then into the flow-through cuvette located inside the spectrophotometer before being discharged into a waste beaker.

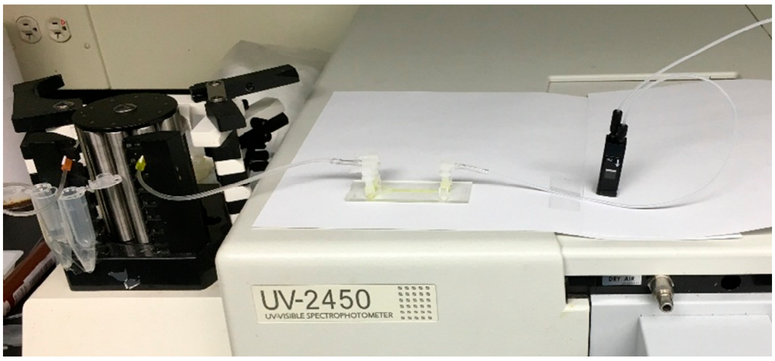

Figure S4. A different view of the experimental setup that was used to analyze the signal transduction process. The flow of solution was controlled by a Gilson MINIPULS® 3, which was used to push solution through the enzymatic flow cell and then into the flow-through cuvette before being discharged into a waste beaker. In this illustration, the flow through cuvette has been plumbed into the system, to illustrate the process.

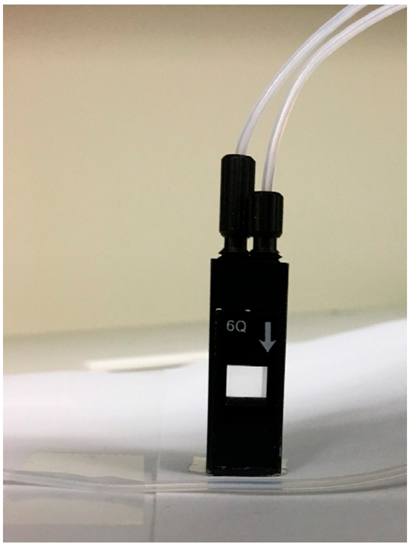

Figure S5. The commercial flow-cuvette connected to the tubing of the flow system. During the measurements the cuvette was placed inside the spectrophotometer.

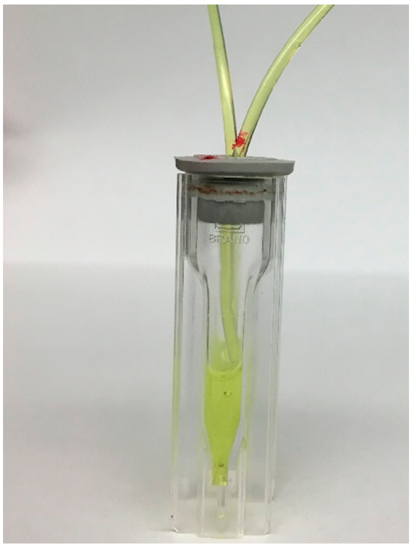

Figure S6. The lab-made flow-cuvette, please note that the volume of solution in the lab-made cuvette is tunable by adjusting the height of the outflow hose. In this illustration, dye is used to make the height of solution visible. During the measurements the cuvette was placed inside the spectrophotometer.

References

1. Fratto, B.E.; Katz, E. Reversible Logic Gates Based on Enzyme-Biocatalyzed Reactions and Realized in Flow Cells: A Modular Approach. *ChemPhysChem* **2015**, *16*, 1405–1415.
2. Fratto, B.E.; Katz, E. Controlled Logic Gates—Switch Gate and Fredkin Gate Based on Enzyme-Biocatalyzed Reactions Realized in Flow Cells. *ChemPhysChem* **2016**, 17, 1046–1053.
